# Efficacy and persistence of long-lasting microbial larvicides against malaria vectors in western Kenya highlands

**DOI:** 10.1186/s13071-018-3009-z

**Published:** 2018-07-31

**Authors:** Samuel C. Kahindi, Simon Muriu, Yahya A. Derua, Xiaoming Wang, Guofa Zhou, Ming-Chieh Lee, Joseph Mwangangi, Harrysone Atieli, Andrew K. Githeko, Guiyun Yan

**Affiliations:** 1 School of Pure and Applied Sciences, Pwani University, Kilifi, Kenya; 2 Climate and Human Health Research Unit, Centre for Global Health Research, Kenya Medical Research Institute, Kisumu, Kenya; 3 Kilimanjaro Christian Medical University College, Tumaini University Makumira, Moshi, Tanzania; 4 Program in Public Health, College of Health Sciences, University of California, Irvine, CA USA; 5 Centre for Geographic Medicine Research-Coast, Kenya Medical Research Institute, Kilifi, Kenya; 6 School of Public Health, Maseno University, Kisumu, Kenya

**Keywords:** *Bacillus thuringiensis* var. *israelensis*, *Bacillus sphaericus*, *Anopheles gambiae* complex, *Anopheles funestus* group

## Abstract

**Background:**

Chemical-based malaria vector control interventions are threatened by the development of insecticide resistance and changes in the behavior of the vectors, and thus require the development of alternative control methods. Bacterial-based larvicides have the potential to target both insecticide resistant and outdoor-biting mosquitoes and are safe to use in the environment. However, the currently available microbial larvicide formulations have a short duration of activity requiring frequent re-applications which increase the cost of control interventions. This study was designed to evaluate the efficacy and duration of activity of two long-lasting formulations of *Bacillus thuringiensis* var. *israelensis* (Bti) and *Bacillus sphaericus* (Bs) (LL3 and FourStar®) under field conditions in western Kenya highlands.

**Methods:**

Three sites were selected for this study in the highlands of western Kenya. In each site, one hundred anopheline larval habitats were selected and assigned to one of three arms: (i) LL3; (ii) FourStar®; and (iii) untreated control larval habitats. Four types of larval habitats were surveyed: abandoned gold mines, drainage canals, fish ponds and non-fish ponds. The habitats were sampled for mosquito larvae by using a standard dipping technique and collected larvae were recorded according to the larval stages of the different *Anopheles* species. The larvicides were applied at manufacturers’ recommended dosage of 1 briquette per 100 square feet. Both treatment and control habitats were sampled for mosquito larvae immediately before treatment (day 0), and then at 24 hours, 3 days and weekly post-treatment for 5 months.

**Results:**

Overall larval density in treatment habitats was significantly reduced after application of the two microbial larvicides as compared to the control habitats. Post-intervention reduction in anopheline larval density by LL3 was 65, 71 and 84% for 1 day, 2 weeks and 4 weeks, respectively. FourStar® reduced anopheline larval density by 60, 66 and 80% for 1 day, 2 weeks and 4 weeks, respectively. Comparisons between the treatments reveal that LL3 and FourStar® were similar in efficacy. A higher reduction in *Anopheles* larval density was observed in the abandoned goldmines, while drainage canals had the lowest reduction.

**Conclusions:**

Both LL3 and FourStar® long-lasting microbial larvicides were effective in reducing immature stages of *An. gambiae* complex and *An. funestus* group species, with significant reductions lasting for three months post-application.

## Background

In recent years, the western Kenya highlands have experienced increased frequencies of malaria epidemics and high transmission rates despite prolonged use of long-lasting insecticidal nets (LLINs) and indoor residual spraying (IRS) for malaria vector control [1–3]. Human population increase and changes in land use such as deforestation and swamp cultivation have been linked with this increase in malaria transmission and local epidemics [2, 4]. Moreover, *Anopheles arabiensis* is increasingly becoming an important malaria vector in the highlands of western Kenya in addition to the well-known *An. gambiae* (*s.s.*) and *An. funestus. Anopheles arabiensis* is difficult to control due to its exophilic and zoophilic tendencies allowing it to survive chemical control better than the highly anthropophagic and endophilic *An. gambiae* (*s.s.*) and *An. funestus* [5, 6]. Moreover, the current chemical-based malaria vector control interventions (LLINs and IRS) are threatened by the development of insecticide resistance and changes in vector biting and resting behavior that require the development of alternative control interventions [7–10].

Mosquito larval control is once again gaining importance owing to the need for integrated vector management approaches to complement measures against adult mosquitoes [11–14]. Biolarvicides based on the Gram-positive spore-forming bacteria, *Bacillus thuringiensis* var. *israelensis* (Bti) and *Bacillus sphaericus* (Bs) can target both insecticide resistant and outdoor biting malaria vectors [15–17]. Several studies in Africa have demonstrated the effectiveness of these bacterial larvicides in granular, powder and tablet formulations in reducing the density of malaria vectors [15, 18–27] and malaria transmission [23, 28]. However, these formulations are of short duration of activity and hence require regular applications which are associated with high operational costs as compared to the longer-lasting chemical-based interventions; hence, their use has not been widely embraced by the national malaria control programs in sub-Saharan Africa [29, 30].

To overcome the problem of low residual activity of the microbial larvicides, new long-lasting formulations that release effective levels of Bti and Bs at the water surface over prolonged periods of time have been developed. However, before deployment of these products for malaria vector control, their effectiveness and persistence have to be verified in natural anopheline breeding habitats in different ecological settings. This study was designed to evaluate two new long-lasting microbial larvicide formulations based on Bti and Bs against different stages of malaria vectors in different aquatic larval habitats in western Kenya highlands.

## Methods

### Study area

**Fig. 1:**
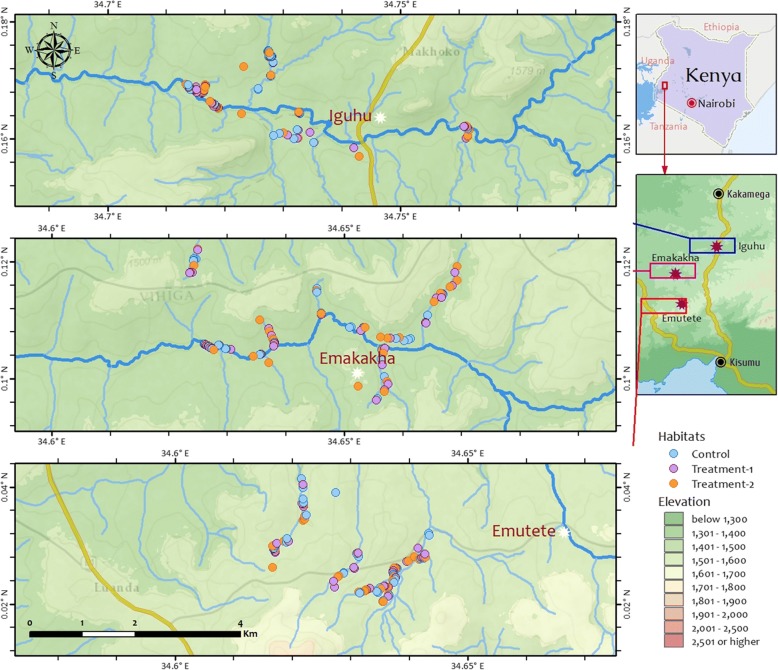
Map of the study area showing distribution of larval habitats

The study was conducted in three villages in the western Kenya highlands, Iguhu (00°09'42"N, 34°45'42"E) in Kakamega County, Emutete (00°01'35"N, 34°37'00"E) and Emakakha (00°06'32"N, 34°39'12"E) in Vihiga County (Fig. 1). All study sites are located in-between Kisumu town and Kakamega town, the two largest cities in western Kenya. Iguhu is located about 15 km south of Kakamega town along the Kisumu-Kakamega road, Emakakha is about 15 km southwest of Iguhu, and Emutete is about 10 km south of Emakakha (Fig. 1). Briefly, the study villages were located in an area of hills and valleys. The hillside mostly comprised comprises maize plantations and a few patches of indigenous forests located along the valleys. The principal occupation of inhabitants of the study villages includes subsistence farmers of maize and some vegetables, and livestock rearing such as cattle, goats, sheep and chickens. The climate consists of a bimodal pattern of rainfall, with a long rainy season from April to June and a short rainy season in October and December. There is no clear dry season in the villages, but usually there is less rainfall from July to September. January and February are the hottest and driest months.

### Larval habitat characterization, selection and randomization

The study villages were surveyed for the presence of anopheline larval habitats and habitats were characterized by type, size, permanence, vegetation coverage and land use types as previously classified [31]. All potential larval habitats identified were enumerated and mapped using a hand held global positioning system (GPS) device before being sampled for mosquito larvae and pupae (Fig. 1). Four types of larval habitats were surveyed, namely abandoned gold mines, drainage canals, non-fish ponds and fish ponds. Abandoned gold mines were pits left after the cessation of gold mining activities while drainage canals were canals used to drain water from farms. Non-fish ponds were natural or man-made relative large water bodies with or without emergent aquatic vegetation. Fish ponds were man-made ponds used for fish farming, and some had fish present during the period of survey.

### Study design

In each of the three villages, 100 anopheline larval breeding habitats were selected for the study and randomized equally into three arms: (i) FourStar®; (ii) LL3; and (iii) untreated control larval habitats. FourStar® is a slow-release briquet formulation with combination of Bti and Bs that the manufacturer claims is effective from 90 to 180 days (Adapco Inc., Sanford, FL, USA). The 30 g formulation consists of 6% by weight of *Bacillus sphaericus* serotype H5a5b strain 2362, 1% by weight of *Bacillus thuringiensis* subspecies *israelensis*, strain BMP 144 and other ingredients that slowly release the bacterial toxins. LL3 has the same Bti/Bs contents as FourStar®, the only difference being that LL3 floats in the water (density approximately 0.99 g/cm^3^) and FourStar® sinks. The briquets were applied at the manufacturers’ recommended dosage of 1 briquet per 100 square feet of larval habitat though hand broadcasting. For habitats with a likelihood of overflow or flow of water during heavy rains, the briquets were fastened to a long loose thin string attached to a pole at the shallow water margins.

### Pre- and post-intervention larval sampling

Selected larval habitats were sampled for mosquito immatures using World Health Organization (WHO) 350 ml standard mosquito dippers. Depending on habitat size, 5 to 20 dips were taken from each larval habitat: 5 dips were undertaken in small larval habitats of ≤ 1 m^2^, 10 dips for medium-sized habitats (2–15 m^2^) and 20 dips for relatively large habitats (> 15 m^2^). The immatures were classified into early instars (1st- and 2nd-stage larvae), late instars (3rd- and 4th-stage larvae) and pupae of different species of *Anopheles*. Both treatment and control larval habitats were sampled on a weekly basis for five weeks before the intervention to obtain baseline information on abundance of mosquito larvae and pupae. The larval habitats were monitored immediately prior to application (day 0) and then on days 1, 3 and weekly after 7 days post-treatment for a period of 5 months.

### Identification of *An. gambiae* complex and *An. funestus* group

In the laboratory, mosquito specimens from each habitat were morphologically identified into *An. gambiae* complex and *An. funestus* group and stored in 80% ethanol. These were later identified as respective sibling species by polymerase chain reaction (PCR) as described previously [32, 33]. In brief, for members of *An. gambiae* complex, PCR reactions were conducted in a final volume of 20 μl consisting of 0.25 μM of each of the five primers, 1:1 DreamTaq Green PCR Master Mix (ThermoScientific, Waltham, USA) and 2 μl of DNA extract. The samples were amplified in Bio-Rad T100 thermo cycler (Bio-Rad, Hercules, CA, USA) and cycling conditions were 95 °C for 5 min followed by 30 cycles of denaturation at 94 °C for 30 s, annealing at 50 °C for 30 s, extension at 72 °C for 30 s and final extension at 72 °C for 10 min.

For *An. funestus* sibling species identification, each PCR run was conducted in a final volume of 25 μl consisting of 0.4 μM of each of the six primers, 1:1 DreamTaq Green PCR Master Mix and 3 μl of extracted DNA. The samples were amplified in a Bio-Rad T100 thermocycler and cycling conditions were 95 °C for 5 min followed by 40 cycles of denaturation at 94 °C for 30 s, annealing at 50 °C for 30 s, extension at 72 °C for 40 s and final extension at 72 °C for 10 min. The amplified DNA for both sibling species of *An. gambiae* and *An. funestus* species complexes were separated based on their fragment size by gel electrophoresis.

### Data analysis

The percentage reduction in the larval and pupal densities was calculated using Mulla’s formula [15] as follows: % reduction = 100 – (C1/T1 × T2/C2) × 100, where C1 and C2 are the counts in control habitats before and after treatment, respectively, and T1 and T2 are the counts in treated habitats before and after treatment, respectively. The differences in abundance of various stages of immature *Anopheles* observed were compared using generalized estimating equations (GEE) based on the Poisson distribution assumption in which baseline and observation time (in weeks) were treated as covariates [34, 35]. The values of the covariates were constant for the repeated elementary observations at each habitat. GEE is considered as a suitable model that handles longitudinal data which is potentially correlated between subsequent observations and thus distorts the independence assumption of the ordinary linear regression model. The correlation between longitudinal observations was tested against four assumptions, i.e. independent (not correlated), exchangeable (fixed correlation), lag 1 autoregression and unstructured (correlations are all different). The Akaike information criterion was used for model parameter estimation and selection. The significant level was *P* = 0.05. If 0.05 < *P* < 0.10, then we defined it as marginally significant, and insignificant otherwise. The models were first run using the interventions against control to evaluate the impact of interventions on the abundance of different species of *Anopheles* immatures, then the two interventions against each other (LL3 *versus* FourStar®) to determine the difference between the two larvicides. Relative reduction in abundance of immature anophelines was calculated as reduction in intervention habitats against control habitats based on pre-intervention observations. Data analysis was conducted by the use of open source language R 3.3.1. For the GEE analysis, the geeglm function in the *geepack* package was used.

## Results

### Descriptive summary

During the 25-week study period, 7,896 samples (1,486 pre-intervention and 6,410 post-intervention) were collected. A total of 43,147 immature anophelines were recorded. Among these, 29,221 were *An. gambiae* (*s.l.*) and 13,926 were *An. funestus* (*s.l.*). During the 5-week pre-intervention period, 49% of habitats had immature *An. gambiae* with an average density of 5.1 individuals per habitat per sampling occasion, and 37% of habitats had immature *An. funestus* with an average density of 2.0 individuals per habitat per sampling occasion. During the 20-week post-intervention period, 27% of habitats had immature *An. gambiae* with an average density of 3.4 individuals per habitat per sampling occasion, 32% of habitats had immature *An. funestus* with an average density of 1.7 individuals per habitat per sampling occasion.

### Impact of FourStar® and LL3 on general *Anopheles* larval density

**Fig. 2:**
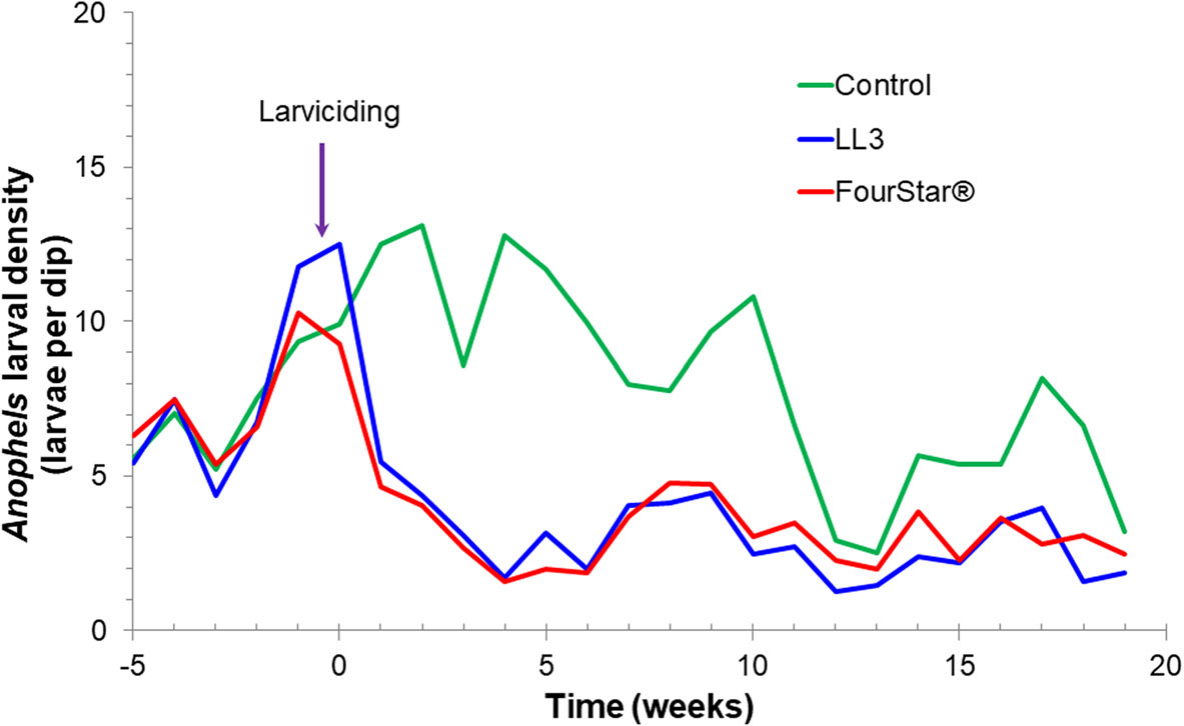
Impact of LL3 and FourStar® larvicides on total *Anopheles* larval population

**Fig. 3:**
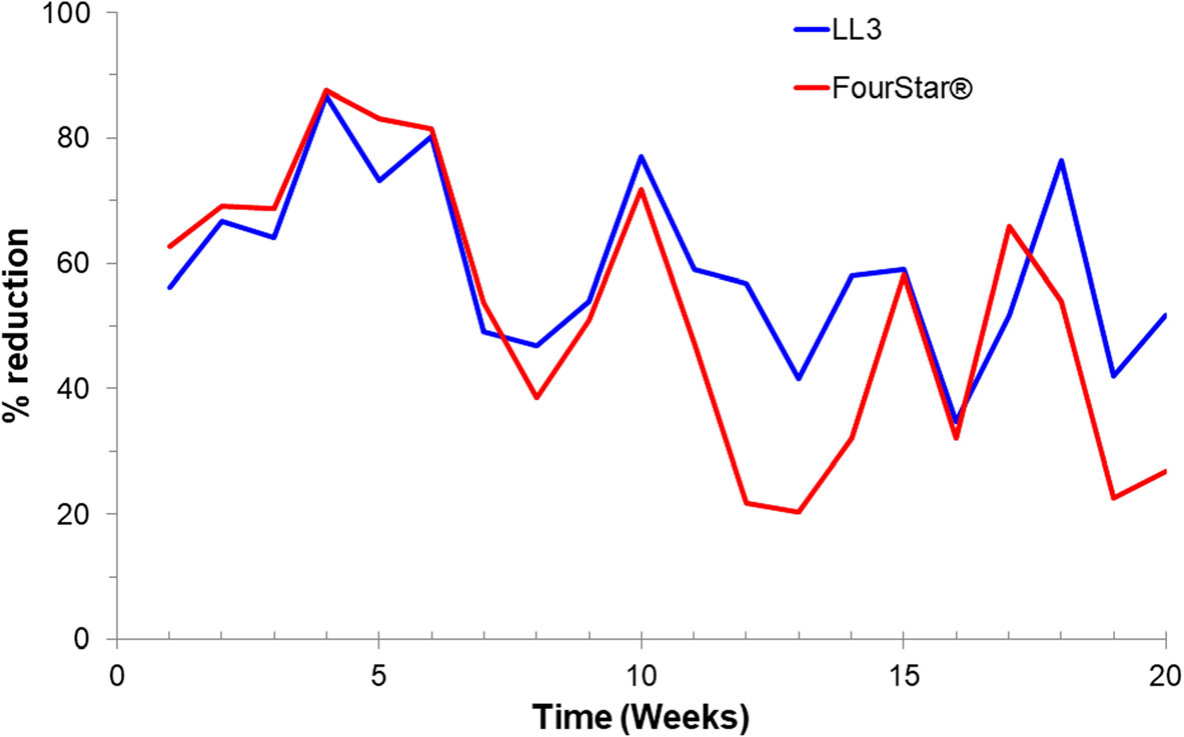
Percentage reduction in total *Anopheles* larval density by LL3 and FourStar® larvicides

During the pre-intervention period, the mean density of *Anopheles* larvae was 8.05, 7.56 and 7.44 larvae per dip for LL3, FourStar® and control arms, respectively (Fig. 2). The mean density of *Anopheles* larvae per dip in treatment habitats (LL3 and FourStar®) and control larval habitats was not significantly different during the pre-intervention period (GEE, *P* > 0.1, Table 1). However, during 20 weeks of post-intervention observation, the mean density of *Anopheles* larvae was significantly reduced to 2.88 and 3.09 for LL3- and FourStar®-treated habitats (Fig. 2), respectively, when compared to the baseline pre-treatment values (GEE, *P* < 0.05, Table 1). On the other hand, *Anopheles* larvae density in control habitats was slightly increased to 7.77 larvae per dip. Post-intervention reduction in anopheline larval density by LL3 was 65, 71 and 84% for 24 hours, 1 week and 4 weeks, respectively. At the same time, FourStar® reduced anopheline larval density by 60, 66 and 80% (Fig. 3, Table 2). By the 5–7 weeks post-application, reduction in larval density averaged 60 and 44% for LL3 and FourStar®, respectively. The reduction in larval density by LL3 remained above 50% for up to 12 weeks (Fig. 3). Overall, the two interventions, LL3 and FourStar® did not show significant difference in reducing immature mosquito densities (Table 2).

**Table 1:** Generalized estimating equations (GEE) analysis of the effect of larviciding on the density of immature mosquitoes, showing the probabilty of the factors

Species	Immature stage or time	Comparing intervention *versus* control		
		Intervention stage^a^	Intervention^b^	Intervention stage × intervention
*An. gambiae* (*s.l.*)	1st- and 2nd-instar larvae	0.011	0.002	0.001
	3rd- and 4th-instar larvae	0.009	<0.001	<0.001
	Pupae	0.952	0.084	0.011
*An. funestus* (*s.l.*)	1st- and 2nd-instar larvae	0.895	0.068	0.008
	3rd- and 4th-instar larvae	<0.001	<0.001	<0.001
	Pupae	0.313	0.890	0.050
Other mosquito species	1st- and 2nd-instar larvae	<0.001	0.002	<0.001
	3rd- and 4th-instar larvae	<0.001	<0.001	<0.001
	Pupae	0.774	0.177	0.001
Total *Anopheles* spp.	By week 12	0.016	<0.001	<0.001
	By week 16	0.197	<0.001	<0.001
	By week 20	<0.001	0.037	<0.001

This analysis compared larviciding against control. Data from the two larvicides were pooled

^a^Intervention stage was classified as pre-intervention *versus* post-intervention

^b^Intervention was classified as larviciding intervention *versus* no-larviciding control

**Table 2:** Generalized estimating equations (GEE) analysis of the effect of LL3 and FourStar® larvicides on the density of immature mosquitoes, showing the probability of the factors

Species	Immature stage or time	Comparing LL3 *versus* FourStar®		
		Intervention stage^a^	Larvicide type^b^	Intervention stage × Larvicide type
*An. gambiae* (*s.l.*)	1st- and 2nd-instar larvae	0.215	0.901	0.918
	3rd- and 4th-instar larvae	<0.001	0.310	0.778
	Pupae	0.001	0.933	0.945
*An. funestus* (*s.l.*)	1st- and 2nd-instar larvae	0.011	0.557	0.348
	3rd- and 4th-instar larvae	0.881	0.409	0.089
	Pupae	0.387	0.809	0.381
Other mosquito species	1st- and 2nd-instar larvae	0.980	0.970	0.710
	3rd- and 4th-instar larvae	<0.001	0.502	0.996
	Pupae	0.002	0.939	0.062
Total *Anopheles* spp.	By week 12	<0.001	0.700	0.950
	By week 16	<0.001	0.995	0.951
	By week 20	0.066	0.536	0.880

^a^Intervention stage was classified as pre-intervention *versus* post-intervention

^b^Larvicide type included LL3 and FourStar®

### Impact of FourStar® and LL3 on density of different larval stages of *An. gambiae* complex and *An. funestus* group

**Fig. 4:**
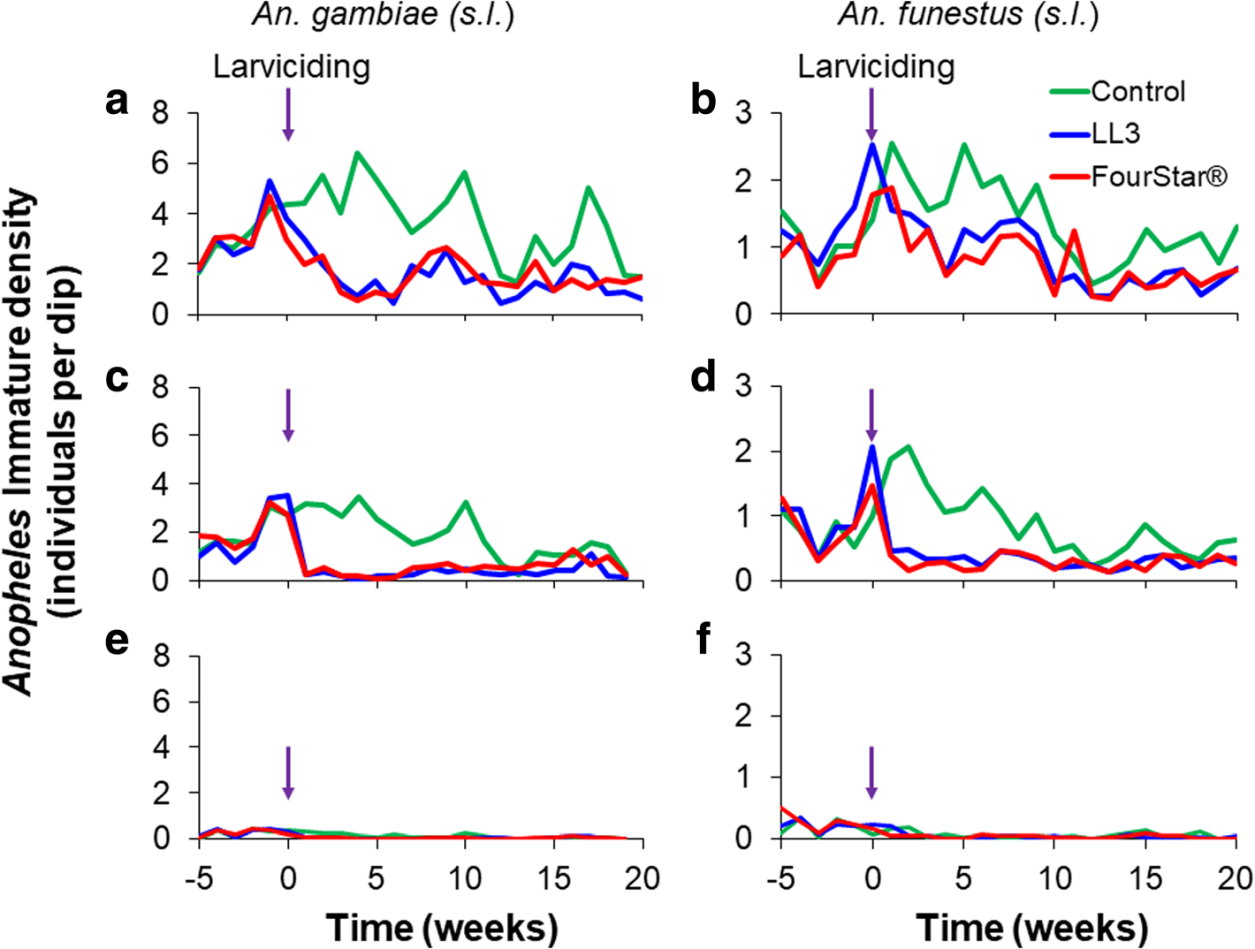
Impact of LL3 and FourStar® larvicides on the density of different immature stages of *Anopheles gambiae* (*s.l.*) and *An. funestus* (*s.l.*). **a** First- and second-instar larvae of *An. gambiae* (*s.l.*). **b** First- and second-instar larvae of *An. funestus* (*s.l.*). **c** Third- and fourth-instar larvae of *An. gambiae* (*s.l.*). **d** Third- and fourth-instar larvae of *An. funestus* (*s.l.*). **e** Pupae of *An. gambiae* (*s.l.*). **f** Pupae of *An. funestus* (*s.l.*). Arrows indicate the time when larvicides were applied

During the baseline, the mean density of *An. gambiae* complex 1st- and 2nd-instar larvae was 3.17, 3.07 and 3.18 larvae per dip for LL3-treated, FourStar®-treated and control habitats, respectively (Fig. 4). The mean larval density for early and late instars of *An. gambiae* complex in the three experimental arms did not differ significantly during this period (GEE, *P* > 0.1, Table 1). However, within the 20-week post-intervention period, the mean density of *An. gambiae* complex early instars was significantly reduced to 1.35 and 1.47 for LL3- and FourStar®-treated habitats, respectively, whereas in control habitats the mean density did not change significantly (GEE, *P* > 0.1). On the other hand, the mean density of late instars of *An. gambiae* complex in the baseline survey was 1.95, 2.12 and 1.95 larvae per dip, for LL3-treated, FourStar®-treated and control habitats, respectively. However, during the 20-week post-intervention period this mean density was significantly reduced to 0.33 and 0.51 larvae per dip (GEE, *P* < 0.05), whereas in control habitats, the mean density did not change significantly (GEE, *P* > 0.1). Likewise, the mean pupal density for *An. gambiae* complex in the pre-intervention period was 0.28, 0.26 and 0.26 pupae per dip in LL3-treated, FourStar®-treated and control larval habitats, respectively. During this period, the mean pupal density between the control and treatment habitats was not significantly different (GEE, *P* > 0.1). However, during the post-treatment period, the mean pupal density for *An. gambiae* complex was significantly reduced to 0.03 and 0.03 pupae per dip in LL3- and FourStar®-treated habitats, respectively (GEE, *P* < 0.05). In all cases, the two interventions, LL3 and FourStar® did not show significant differences in immature mosquito densities (Table 2).

For the *An. funestus* group, the mean larval density for early instars during the baseline period was 1.40, 1.00 and 1.11 larvae per dip in LL3-treated, FourStar®-treated and control habitats, respectively, while for late instars it was 1.04, 0.88 and 0.78 larvae per dip, respectively (Fig. 4). During the 20-week post-intervention period, the mean larval density of *An. funestus* group was significantly reduced in treatment habitats to 0.84 and 0.77 larva per dip for early instars and 0.31 and 0.29 larvae per dip for late instars in LL3- and FourStar®-treated larval habitats, respectively (GEE, *P* < 0.05). The mean larval density for early and late instars of *An. funestus* group in control habitats did not change significantly from the baseline values, and was maintained at 1.38 and 0.82 larvae per dip, respectively (GEE, *P* > 0.1). The mean pupal density for *An. funestus* group species in the pre-intervention period was 0.21, 0.25 and 0.17 pupae per dip in LL3-treated, FourStar®-treated and control habitats, respectively. The mean pupal density in treated and control habitats pre-treatment did not differ significantly (GEE, *P* > 0.1). However, during the post treatment period, there was only a marginal reduction in *An. funestus* group mean pupal density in both the LL3 and FourStar® treatments (Fig. 4). In all cases, the two interventions, LL3 and FourStar® did not show significant difference in reducing immature mosquito densities (Table 2).

### Impact of FourStar® and LL3 in different larval habitat types

**Fig. 5:**
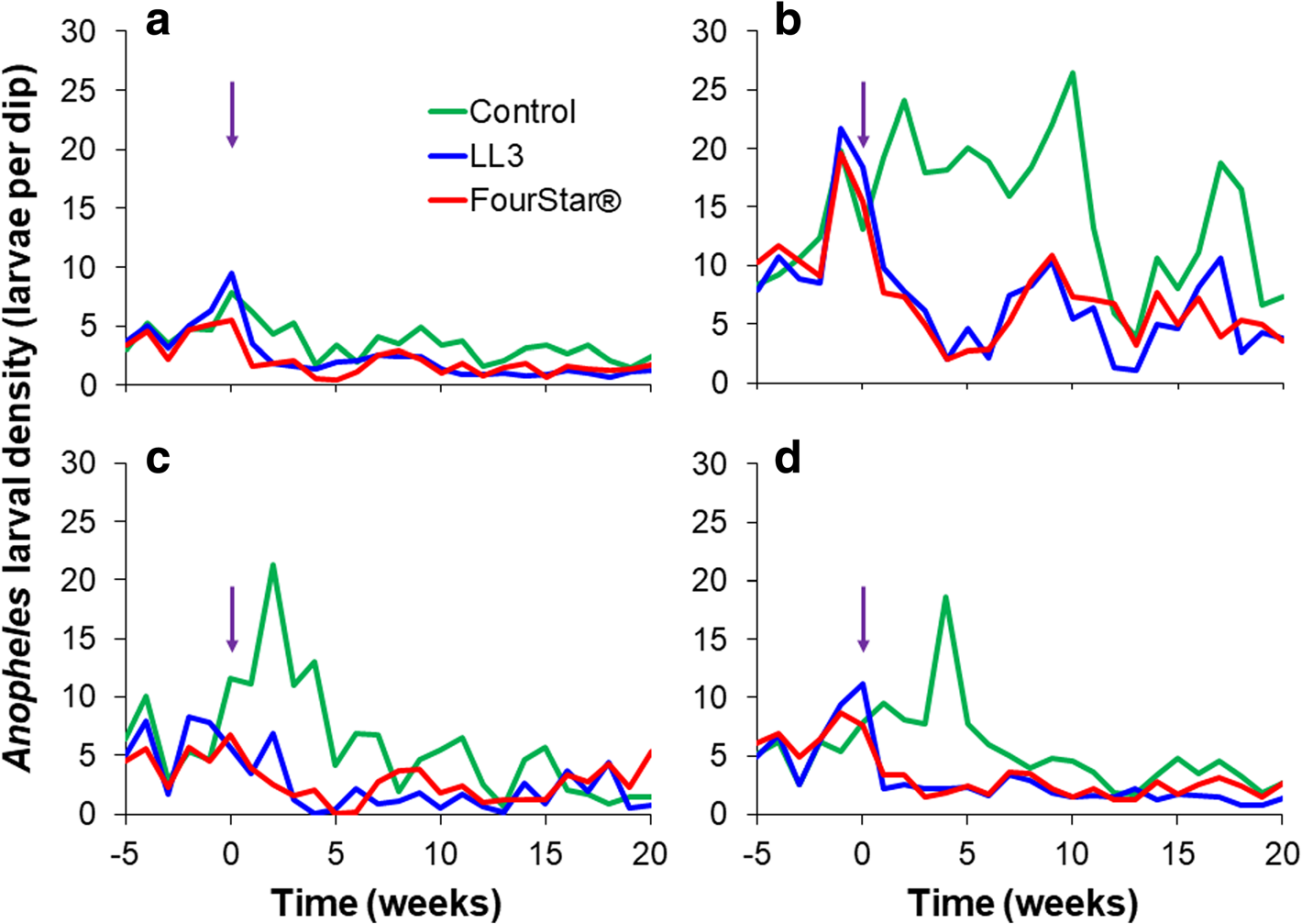
Impact of LL3 and FourStar® larvicides on *Anopheles* larval density in different types of habitats. **a** Drainage canals; **b** Abandoned goldmines; **c** Fish ponds; and **d** Non-fish ponds. Arrows indicate the time when larvicides were applied

During the study, four types of aquatic habitats were found to be common breeding sites of *Anopheles* larvae: abandoned goldmines, drainage canals, non-fish ponds and fish ponds (Fig. 5). In the abandoned goldmines, mean *Anopheles* larval density in the control habitats during the pre-intervention period was 12.25 and increased to 15.17 larvae per dip during the intervention period. In the treated habitats, the baseline mean *Anopheles* larvae density was 12.69 and 12.76 for LL3- and FourStar®-treated habitats, respectively (Fig. 5). However, during the post-intervention period, this decreased significantly to an average of 5.61 and 5.73 larvae per dip for LL3- and FourStar®-treated habitats, respectively (GEE, *P* < 0.05). This reduction in the mean *Anopheles* larval density remained significantly low compared to the baseline pre-intervention values for up to 12 weeks.

For drainage canals, the *Anopheles* larval productivity was reduced significantly after larviciding from the baseline pre-intervention values of 4.87 to 1.57 larvae per dip after treatment in LL3 and 5.46 to 1.55 larvae per dip after treatment in FourStar®-treated habitats (GEE, *P* < 0.05). The trend was similar in the fish ponds, where the pre-intervention period *Anopheles* larvae density was 6.79 and 6.05 larvae per dip for LL3- and FourStar®-treated habitats, respectively, but decreased significantly to 1.75 and 2.34 larvae per dip, respectively, during the post-intervention period (GEE, *P* < 0.05). In the non-fish ponds, mean *Anopheles* larval density during the pre-intervention period was 5.56, 6.84 and 6.77 in control, LL3- and FourStar®-treated habitats, respectively. Twenty-four hours post-intervention period mean *Anopheles* larval density decreased significantly to 1.84 and 2.31 larvae per dip in LL3- and FourStar®-treated habitats, respectively (GEE, *P* < 0.05), while in the control the density was not significantly different from the baseline value (Fig. 5). Overall, the highest reduction in *Anopheles* larval density was observed in the abandoned goldmines, while drainage canals had the lowest reduction.

### Species identity of larval specimens of *An. gambiae* complex and *An. funestus* group

A total of 366 *An. gambiae* complex and 237 *An. funestus* group larval specimens were morphologically identified and then processed for sibling species identity using PCR technique. In the *An. gambiae* complex, *An. gambiae *(*s.s.*) were the predominant sibling species, accounting for 70.8% of the identified specimens. On the other hand, *An. funestus* (*s.s.*) accounted for 73.6% of the identified sibling species of the *An. funestus* group (Table 3).

**Table 3:** PCR testing of larval specimens for their identity in the sibling species of the *Anopheles gambiae* complex and *An. funestus* group

Malaria vector	No. tested	PCR positive^a^	Sibling species identified	Total (%)
*An. gambiae* complex	366	305	*An. gambiae* (*s.s.*)	216 (70.8)
			*An. arabiensis*	89 (29.2)
*An. funestus* group	237	174	*An. funestus* (*s.s.*)	128 (73.6)
			*An. leesoni*	46 (26.4)

^a^61 (16.7%) and 63 (26.6%) specimens identified morphologically as *An. gambiae* complex and *An. funestus* group, respectively, did not amplify

## Discussion

Mosquito larval control has been found to be effective in lowering malaria transmission as it targets both indoor and outdoor biting mosquito species [23, 36]. Larviciding with microbial larvicides has proved to be effective in mosquito control but with low residual activity requiring repeated applications with high cost implications [29, 30]. Recently, long-lasting microbial larvicide formulations with potential for sustained larval control have been developed to overcome the problem of low persistence, thereby improving cost-effectiveness. The current study was designed to test two long-lasting formulations of microbial larvicides (FourStar® and LL3) against different stages of malaria vectors in different aquatic larval habitats in the highlands of western Kenya. In the study area, the malaria vectors *An. gambiae* (*s.s.*) and *An. funestus* (*s.s.*) were the predominant sibling species of the *An. gambiae* complex and *An. funestus* group, respectively.

Results showed that both FourStar® and LL3 long-lasting microbial larvicides were effective in reducing the density of *Anopheles* larval populations, as shown by the significant differences observed between the treated and control larval habitats during the post-intervention period. This was not the case during the baseline survey period where the larval density between the treatment and control habitats was broadly similar, but this trend changed markedly after application of FourStar® and LL3. The observed fluctuations in *Anopheles* larval densities after application of microbial larvicides could be due to the effect of heavy rains experienced during the study period. The heavy rains results in flushing of the floating toxins and thereby reducing the impact of the larvicides. The impact of both larvicides in reducing *Anopheles* larval densities was clearly observed during the first twelve weeks post-application and this is consistent with another study performed previously in the same area [36].

The findings have shown that the two long-lasting microbial larvicides tested were equally effective in reducing the density of both the early and late larval stages of *An. gambiae* complex and *An. funestus* group. This reduction was, however, higher in species of *An. gambiae* complex as compared to *An. funestus* group. This could be due to the fact that *An. gambiae* complex were found in open sunlit temporary to semi-permanent habitats with less vegetation cover where the larvicidal toxins can disperse better with high chances of being consumed by the target larvae. For the case of *An. funestus* group, larval habitats were more permanent with high levels of vegetation which may interfere with the spread of the toxins and hence reduce their availability to the target larvae.

In the current study, reductions in pupal densities were significant in *An. gambiae* complex while in *An. funestus* group they were only marginal. Reduced activity of long-lasting microbial larvicides against pupae of *An. funestus* group observed in the current study agreed with findings of a recent previous study performed in the same area [36]. Since a reduction in pupal densities is a good indicator of reduced adult emergence, low reductions in pupal of *An. funestus* group may indicate low activity of the long-lasting microbial larvicides against this important malaria vector. Analysis of larval and pupal reductions in different larval habitat types studied indicated a reduced persistence of long-lasting microbial larvicides in some larval habitats. The larvicides were more effective in reducing larval densities in the abandoned goldmines, non-fish ponds and fish ponds, which were mainly stagnant water breeding habitats. Efficacy was, however, relatively low in drainage canals and this may be due to the fact that drainage may allow water to flow during the rains and wash away floating larvicide toxins and hence reduce the effectiveness of the intervention. This finding is in line with a study at the Kenyan coast [26] which showed reduced persistence of Bti/Bs in drainage canals. Despite the observed effectiveness of LL3 and FourStar® larvicides in reducing larvae of malaria vectors, their activity varied with vector species and habitat types. Thus, based on our findings, habitat type is an important criterion when considering LL3 and FourStar® for integrated vector management operations. The study demonstrates that the two larvicides are very target specific and environmentally benign and can be applied in various water bodies without fear of detrimental effects on non-target organisms.

## Conclusions

This study showed that both LL3 and FourStar® long-lasting microbial larvicides were effective in reducing larvae and pupae of *An. gambiae* complex and *An. funestus* group mosquitoes for more than three months. Based on marginal levels of immature reduction observed, these long-lasting microbial larvicides are not suitable for use as stand-alone interventions, and should be integrated with the existing tools for malaria vector control.

## Abbreviations

Bs: *Bacillus sphaericus*
Bti: *Bacillus thuringiensis* var. *israelensis*
GEE: Generalized estimating equations
GPS: Global positioning system
IRS: Indoor residual spraying
LLINs: Long-lasting insecticidal nets
PCR: Polymerase chain reaction
WHO: World Health Organization
